# Drug Repurposing in Cancer Therapy: Influence of Patient’s Genetic Background in Breast Cancer Treatment

**DOI:** 10.3390/ijms23084280

**Published:** 2022-04-14

**Authors:** Rafaela Rodrigues, Diana Duarte, Nuno Vale

**Affiliations:** 1 OncoPharma Research Group, Center for Health Technology and Services Research (CINTESIS), Rua Dr. Plácido da Costa, 4200-450 Porto, Portugal; rafaela24sofia@hotmail.com (R.R.); dianaduarte29@gmail.com (D.D.); 2 Faculty of Pharmacy of University of Porto, Rua Jorge Viterbo Ferreira, 228, 4050-313 Porto, Portugal; 3 Department of Community Medicine, Health Information and Decision (MEDCIDS), Faculty of Medicine, University of Porto, Al. Prof. Hernâni Monteiro, 4200-319 Porto, Portugal; 4 Associate Laboratory RISE–Health Research Network, Faculty of Medicine, University of Porto, Al. Prof. Hernâni Monteiro, 4200-319 Porto, Portugal

**Keywords:** repurposed drugs, genetic influence, anticancer drugs, cancer drug resistance, pharmacology, therapeutic strategies, personalized medicine

## Abstract

Cancer is among the leading causes of death worldwide and it is estimated that in 2040 more than 29 million people will be diagnosed with some type of cancer. The most prevalent type of cancer in women, worldwide, is breast cancer, a type of cancer associated with a huge death rate. This high mortality is mainly a consequence of the development of drug resistance, which is one of the major challenges to overcome in breast cancer treatment. As a result, research has been focused on finding novel therapeutical weapons, specifically ones that allow for a personalized treatment, based on patients’ characteristics. Although the scientific community has been concerned about guaranteeing the quality of life of cancer patients, researchers are also aware of the increasing costs related to cancer treatment, and efforts have been made to find alternatives to the development of new drugs. The development of new drugs presents some disadvantages as it is a multistep process that is time- and money-consuming, involving clinical trials that commonly fail in the initial phases. A strategy to overcome these disadvantages is drug repurposing. In this review, we focused on describing potential repurposed drugs in the therapy of breast cancer, considering their pharmacogenomic profile, to assess the relationship between patients’ genetic variations and their response to a certain therapy. This review supports the need for the development of further fundamental studies in this area, in order to investigate and expand the knowledge of the currently used and novel potential drugs to treat breast cancer. Future clinical trials should focus on developing strategies to group cancer patients according to their clinical and biological similarities and to discover new potential targets, to enable cancer therapy to be more effective and personalized.

## 1. Introduction

**Figure 1:**
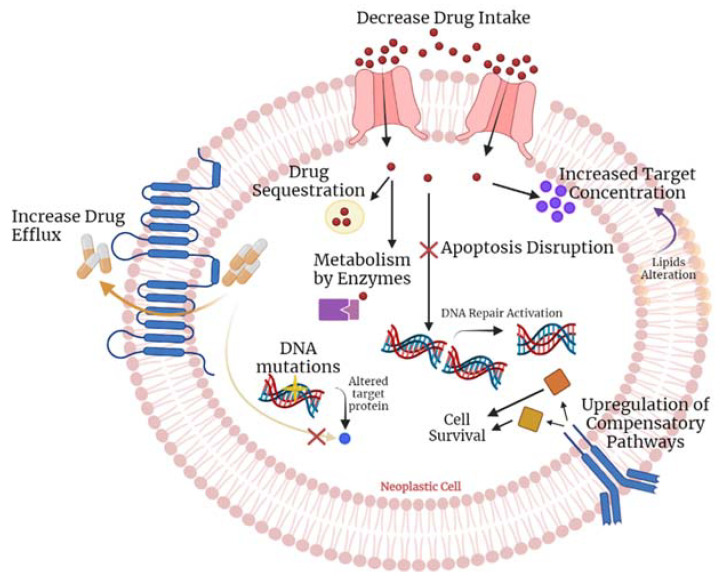
Molecular mechanisms involved in the development of multiple drug resistance by the neoplastic cells. Briefly, increase drug efflux, decrease drug intake, drug sequestration, and drug metabolism by enzymes, are the mechanisms responsible for the decreased drug availability in the cells. Additionally, through increased target concentration, apoptosis disruption, DNA mutations, and upregulation of compensatory pathways, cancer cells can surpass the anti-cancer action of the drug. Figure created using BioRender.

Despite the huge efforts that have been made in the improvement of cancer therapies over the years, cancer is still one of the leading causes of death worldwide. Its high incidence and mortality rates make this disease one of the major health concerns globally [1]. Although there is a large number of therapeutic strategies available to combat this disease, the current therapeutic schemes are usually accompanied by the development of drug resistance by the tumour cells, which results in a decrease in the antitumor efficacy of the therapeutic agents (Figure 1). This reflects the urgent need for the development of novel therapies or new anti-cancer drugs to overcome this phenomenon [2,3,4]. Nevertheless, the process of developing new drugs is a significantly expensive multistep process, that involves the drug design and synthesis, as well as its repeated testing in animal models, to assure accurate safety and efficacy. Further clinical evaluation is also required to establish proper dosing, safety, and efficacy, and the translational process into clinics is only completed after several regulatory, approval, and commercial considerations [5]. To overcome these issues, researchers have focused on an alternative strategy, drug repurposing, that uses already licensed molecules for other diseases besides their original indication. Contrary to the *de novo* drug development, drug repurposing has numerous advantages that include higher efficiency, lower time and financial expenditure, and reduced risk of failure. It also takes advantage of the knowledge of the drug’s mechanisms of action, contributing to the major efficiency of the clinical translational process [6,7]. Notwithstanding, this repurpose process needs further advances in the field of the drug evaluation by in silico approaches, because despite the fact it is associated with many advantages, providing new insights regarding pharmacokinetic and pharmacodynamic properties, indeed, it is not enough to assess all the information needed for the drug to receive approval for other diseases, as cited by Sonaye and colleagues [8]. Recently, there have been reports defending that in line with multi-omics analysis, machine learning approaches can give insights regarding the molecular docking of such a drug with improved accuracy to better understand drug-target interactions. This is easily implemented currently, using available platforms such as TensorFlow [9,10]. However, the quantity and quality of available data is a limitation of machine learning applications in the drug repurposing field. Despite the fact that this theme is out of the scope of this manuscript, it is important to highlight that the computational methods correlated to multi-omics analysis will provide a vision of more personalized treatment in oncology with repurposing drugs in the future [9,11,12].

In 2010, the estimated financial effort of all countries concerning cancer-related treatment was around $1.16 trillion [13]. This high value can be justified by the economic expenses involved in the development of a new drug for cancer treatment, which are estimated to be around 161–1800 million dollars. In addition, this is a process that takes about 11–14 years, with a success rate of less than 6% [14,15]. On the other hand, the drug repurposing process only takes approximately 6.5 years, with associated costs of around $300 million [16]. This strategy is further explored in the next section.

In this review, we investigated how drug repurposing can help to overcome the current challenges of cancer therapy, considering the cancer patients’ genetic heterogenicity and the complexity of the disease, to offer personalized medicine to each patient. We first started to describe the potential of drug repurposing as a new window of opportunity for cancer therapy, and specifically its application for precision medicine. We next selected some repurposed drugs for breast cancer and have explored the biomarkers associated with their response and their reported outcomes, relating them to the genetic background of patients. Herein, we also highlight the urgent need for the molecular stratification of the patients in order to group the responsive individuals, to improve the efficacy of the drug repurposing approach in breast cancer [17].

## 2. Drug Repurposing: New Window of Opportunity to Treat Cancer

Drug repurposing, also known as drug repositioning, is a strategy that explores alternative uses of an already approved drug, for other diseases besides its original indication. It has been proposed by several authors as an alternative way to increase the number of therapeutic weapons available for cancer treatment and presents many advantages compared to *de novo* cancer drug development. There is no need for extensive studies because the drug’s pharmacokinetic and pharmacodynamic profiles are already full characterized, which shortens the translational process and consequently lowers the associated costs, contributing to a major success of the process [2,5,7]. Currently, according to Kaushik and colleagues, this strategy involves some critical steps that researchers must follow to identify the repurposing potential of a specific drug, involving not only pre-clinical approaches such as in silico, in vitro, or in vivo studies but also clinical observations and epidemiological studies [18,19,20]. Therefore, by identifying the anti-neoplastic effects of a drug and studying its targeted molecular pathways, it is possible to find promising candidates for drug repurposing for anti-cancer therapy. Frequently, a repurposed drug can also have unknown molecular mechanisms that interact with any pathway that is involved in the cancer hallmarks proposed by Hanahan in 2000 (updated in 2021), the so-called “off-target” effects of the therapeutic agents, which can result in unexpected anti-tumoral effects [20,21,22]. Another way to find promising candidates for drug repurposing is by studying their modulation of gene expression associated with a specific cancer profile. Sometimes, genes can be up- or down-regulated in some types of cancer and drugs that can influence their expression can be useful for oncology treatment [22,23].

### 2.1. Drug Repurposing in a Medicine of Precision Point of View

There are also some challenges to face in drug repurposing therapy [23]. Repurposed drugs can be used as monotherapy, as chemopreventive agents, or to enhance other chemotherapeutic agents’ roles. They can also be used to control the adverse effects of other drugs or as an adjuvant treatment, to avoid tumour recurrence. They can be combined with other drugs, to target different oncogenic pathways or to act synergistically in eradicating the tumour. Their use in monotherapy increases the risk of the development of drug resistance [24,25]. A drug combination therapeutic scheme is, usually, more successful than monotherapy since each of the therapeutic agents can act in distinct pathways and exacerbate the final anti-tumoral effect [2,18]. Nevertheless, despite the enhanced anticancer effects, a multidrug scheme of treatment can result in increased adverse side effects, caused by drug-drug interactions, that can impair the treatment [26]. To avoid these complications, it is important to maintain a follow-up of the patients and to perform more extensive molecular studies. Moreover, one of the major challenges to overcome in cancer treatment is the heterogeneity between the responses in oncological patients. This means that the same drug can have more or less success due to patients’ heterogeneity, leading to the need for more personalized treatment. In addition, it is important to highlight that tumour heterogeneity is the major factor contributing to tumour resistance mechanisms. Therefore, besides having new anticancer drugs available for the patient’s treatment, it is also important to change the way these patients are treated, focusing on patient’s intra- and inter-tumour heterogeneity, as a universal treatment model may not adequately address cancer patients’ genetic variations [26,27].

Recently, personalized medicine, accompanied by the progress of genomics, has become a hot topic concerning the search for effective and innovative treatment options for each patient. Due to a patient’s genetic variations, the mode of how the drug is absorbed, metabolized, distributed, and excreted (pharmacokinetics), and the drug’s role in the body (pharmacodynamic) can be different among patients and contribute to different outcomes in drug therapy [28,29]. Therefore, to treat a patient with a certain drug, one must consider pharmacogenetics, a tool used in medicine of precision, that allows the prediction of the response to a therapeutic scheme based on specific genetic biomarkers. This tool helps to understand if there is tumour sensitivity to the drug or even if the patient presents some polymorphism that could give rise to unexpected toxicity or response [28,30]. Thus, it is important to perform extensive genomic studies that help to build a mutational profile of the patients, allowing to group patients according to their molecular features. This will help to select the appropriate therapeutic regimens based on the patients’ genetic background [29,31].

### 2.2. Repurposed Drugs in Breast Cancer Treatment: Biomarkers Associated with Different Outcomes

In this review, we have focused on some repurposed drugs used in breast cancer and reported the biomarkers associated with the different outcomes, such as the anticancer response or toxicity. These biomarkers can be used, in the future, to group breast cancer patients based on their genetic profile and personalize their treatment. Currently, some of the potential repurposed drugs in oncology include antibiotics, non-steroidal anti-inflammatory, cardiovascular, antidepressants, and antipsychotic drugs [30,32]. Table 1 describes some of the repurposed drugs already used in breast cancer therapy and the biomarkers related to the drug’s outcomes. These biomarkers must be considered in future studies to allow the accurate translational applicability of these drugs in personalized clinical practice. To the best of our knowledge, these repurposed drugs are the ones associated with the major relevant evidence concerning their effect depending on patient genetic variations. Thus, they are further explored in the following paragraphs and must be a target of future studies, involving a higher number of breast cancer patients and stratification groups based on their genetic profile.

Doxorubicin belongs to the class of anthracyclines, a subclass of antibiotics, which used to be extracted from *Streptomyces peucetius*. Its approval for medical use occurred in 1974, but only 20 years later its relevance for breast cancer treatment was clarified [31,32]. Its mechanism of action is through DNA intercalation, giving rise to DNA strand breaks, and through the disruption of topoisomerase-II-mediated DNA repair, inhibiting the DNA replication mechanism. This drug is commonly used as a chemotherapeutic agent in breast cancer treatment and other malignancies, despite presenting patient-dependent tumour response and being associated with high toxicity [29,30,31,32]. Indeed, cardiotoxicity is one of the most important parameters to consider when administering doxorubicin, as several studies suggest that the degree of toxicity is influenced by the genetic variability of the patients [31,32,34,35]. Cardiotoxicity induced by doxorubicin can be attributed to the fact that it functions as a substrate of protein importers in both breast tissues and cardiac muscle [34,36]. Todorova and colleagues investigated human leukocyte antigen (HLA) region single nucleotide polymorphisms (SNPs) and proposed that polymorphisms in this region of the genome could predispose individuals to doxorubicin mediated cardiotoxicity, associated with immune and inflammatory dysregulation [31,34]. In addition, other studies suggest that the evaluation of cytokine profile must be performed, in order to be used as a biomarker to assess the cardio-sensitivity of the patients prior to doxorubicin treatment [37]. Other studies suggest that curcumin may have a protective role against doxorubicin toxicity and therefore further investigation should aim to evaluate if its use in combination with doxorubicin is beneficial compared to monotherapy [32,35]. Furthermore, there are reports defending that *CREB3L1* higher expression is correlated with a better response to doxorubicin treatment in triple-negative breast cancer, making it an interesting biomarker to analyse before doxorubicin administration [36,37,38,39]. These results evidence the urgent need for the development of new approaches to help understand the biomarkers’ clinical value to guide a personalized cancer treatment and to evaluate the patients’ risk based on their genetic profile.

Cyclophosphamide is an alkylating agent that has proven efficacy in the modulation of the immune system. It inhibits the suppressive regulatory T cells and enhances the effector T cells in the tumour microenvironment [31]. This drug has been repurposed to treat breast cancer and other malignancies. This drug assumes different functions depending on its dose: at lower concentrations it modulates the immune system, while at higher concentrations it acts as an alkylating agent, causing cancer and lymphoid cell death [40]. This chemotherapeutic agent depends on bioactivation by cytochrome P450 (CYP) enzymes. These enzymes are encoded by several genes, especially *CYP2B6* and *CYP2C19*, which are known to be polymorphic. Some of these genetic variants could lead to function alteration or even loss of function, reflecting the need for more comprehensive studies to understand the roles of several variations of these genes in the response to therapy. Some reports defend that the genetic polymorphisms in those genes, *CYP2B6* (rs12721655, rs3745274, *1, *6) and *CYP2C19* (rs4244285, *1/*17/*2), are related to the efficacy of cyclophosphamide treatment in breast cancer due to its influence on bioactivation of the drug [41,42]. These results support that cancer patients must be genotyped for these gene SNPs prior to cyclophosphamide administration, as these variants are related to the metabolic conversion of the drug into its bioactive form.

Everolimus is a repurposed drug that was initially approved for renal cancer (2009), then approved to suppress the immune system during renal transplants in 2010 and finally approved for the treatment of pancreatic cancer (2011) [31]. Everolimus shows its anti-neoplastic role through its influence on the mTOR pathway and it is currently used in anastrozole- and letrozole-resistant metastatic breast cancer treatment. It received its approval for this type of cancer by the Food and Drug Administration (FDA) in 2012. More recently, this drug was also included in the phase III clinical trial ‘Breast Cancer Trial of Oral Everolimus-2 (BOLERO-2)′, where it was studied its combination with exemestane for the treatment of HR+, HER2 advanced metastatic cancers that are resistant to letrozole or anastrozole [43,44]. Like many other drugs, everolimus is metabolized by cytochrome enzymes. Some of the genes involved in its metabolism, such as the *CYP3A4* (rs35599367), have been linked to a lower drug metabolic rate, although more studies are still needed to support this. Moreover, its pharmacokinetic profile varies according to the genetic variants of some genes, particularly those involved in PI3K/AKT/mTOR pathway, as well as the gene encoding its protein transporter (ABCB1). Some reports link the toxicity of everolimus to several SNPs in PI3K/AKT/mTOR pathway genes, as well as to *ABCB1* (rs1045642), associated with the development of mucositis, *ABCB1* (rs2032582), associated with lymphopenia, *PIK3R1* (rs10515074), associated with hyperglycemia and leucopenia, and *RAPTOR* (rs9906827), associated with pneumonitis [45,46]. Therefore, clinicians must be aware of these genetic variants to provide adequate treatments for each patient, in order to avoid the toxicities associated with this drug.

**Figure 2:**
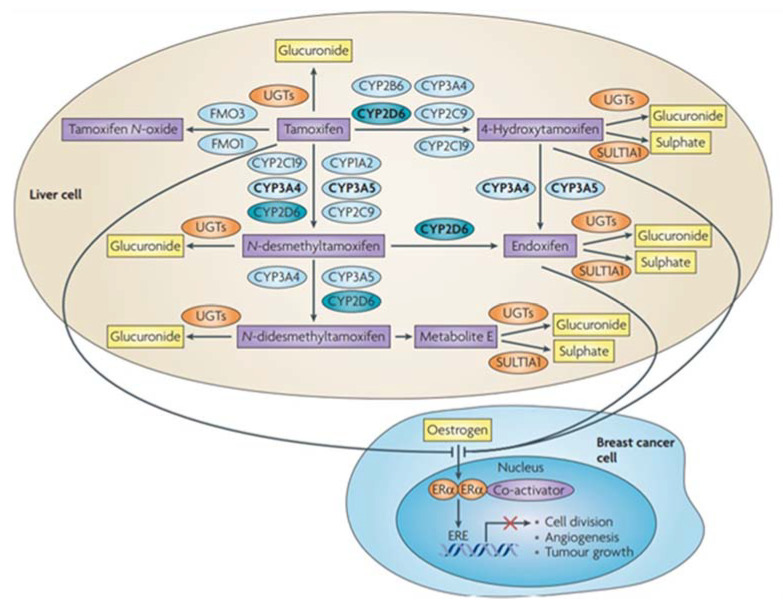
Influence of *CYP2D6* polymorphisms and other cytochrome P450 enzymes in the metabolism of tamoxifen, an antineoplastic drug used in breast cancer, into its active and non-active forms. Reproduced from [45].

Tamoxifen is a selective estrogen receptor modulator, and it was first purposed for the Albright syndrome, as well as to induce ovulation [29,31]. As a competitive inhibitor of estradiol, it can bind also to the estrogen receptors and display anti-neoplastic functions in the mammary tissue. For this reason, several decades ago, tamoxifen was proposed as an adjuvant treatment for hormone receptor-positive breast cancer [42,47]. The major challenge when using tamoxifen is the tumour resistance to the therapy and its adverse effects on the liver [41,48]. The mechanisms of drug resistance could be attributed to pharmacologic reasons, detailed in Figure 2: tamoxifen is converted into its main active form, endoxifen, through the action mediated by genes of the cytochrome P450 family, specifically, *CYP2D6*, which has a myriad of genetic polymorphisms associated with it [47]. A lot of studies have been developed to assess the influence of the genetic variants of *CYP2D6* in tumour resistance. However, to date, there is no clear association due to the conflicting findings [49,50,51]. For that reason, the association between SNPs in *CYP2D6* and the mechanisms of drug resistance and side effects observed with the treatment with tamoxifen must be clarified to link the genotyping of this gene with the disease outcomes [48]. Recently, a clear association between SNPs in the *CYP2D6* and the metabolic rate of tamoxifen was established. This supports the importance of genotyping this gene to allow an adjustment of drug dosages to increase the efficacy of the therapy, or in the case of poor metabolizers, indicates that this drug is not adequate and another one must be selected [42]. Additionally, this study also describes rs4646 SNP of *CYP19A1* as being associated with the effectiveness of tamoxifen.

Anastrazole is a repurposed drug that was originally used for ovary stimulation and the induction of ovulation in infertile females or women with polycystic ovary syndrome. This drug functions as an aromatase inhibitor, reducing estrogen production and is therefore used in ER+ breast cancer treatment [31]. However, recent findings warn of the eventuality of this drug becoming a ligand for estrogen receptors, binding to ERα and activating the downstream pathway of malignant growth [52]. Consequently, it is of great importance to perform further studies to clarify the mechanisms of anastrozole, to provide clear evidence when this drug becomes a ligand of estrogen receptor, allowing to design adequate treatments and improve clinical outcomes. Some reports defend that a genetic variant in *CSMD1*, associated with increased expression of CSMD1 and CYP19 proteins, sensitizes malignant cells to be responders of anastrozole. Indeed, it has been reported that patients who carry rs4646 SNP in *CYP19A1* have a good response to anastrozole [38,42]. Thus, predicting which tumours are more sensitive to this therapeutic strategy is mandatory for adequate oncologic treatment. According to the previous studies, *CSMD1* and *CYP19A1* SNPs could, in fact, be used as predictive markers of anastrozole response in breast cancer [50,53].

Paclitaxel is another repurposed drug whose initial use was addressed to arterial restenosis. It was isolated for the first time in 1971 from pacific yew. In cancer, its first use was in ovarian cancer, in 1992 [31]. Currently, paclitaxel is used as a neoadjuvant or adjuvant treatment of breast cancer, as well as ovarian cancer. Its anti-tumoral mechanism is by the inhibition of mitosis of the cells, decreasing the proliferation rate of cancer cells [29,31]. The tumour response rate to paclitaxel has a largely associated dispersion, attributed to the genetic variation of the patients. In silico studies have reported some SNPs as potential predictive biomarkers of tumour insensitivity to paclitaxel, located on *LPHN2*, *ROBO1*, *SNTG1*, and *GRIK1*. Currently, these results are still under research for validation to proceed with translational application [54,55].

Another well-known drug repurposed to treat triple-negative breast cancer is aspirin [33]. Aspirin is an antiplatelet agent that was initially used for cardiovascular diseases, but its clinical use was further expanded with the discovery of its anti-tumoral effect. Some studies refer that aspirin regular use is linked to a decrease in breast cancer risk [56]. Importantly, polymorphisms in *PIK3CA* have been reported as being related to different effects of aspirin in breast cancer treatment [57]. This is supported by the fact that aspirin can suppress the growth in breast cancer cells that have a mutation in this gene, through the activation of AMPK and mTORC1 inhibition signalling pathways [58]. Therefore, Henry et al. suggest that breast cancer can be potentially treated using a combinatory therapy between aspirin and PI3K inhibitors. Nevertheless, Henry and colleagues defend that for this to be effective, it is essential to previously stratify these patients according to their genetic profile regarding PI3K gene, to find potential responders to this therapy [58].

## 3. Discussion of the Future Directions in Cancer Treatment concerning Drug Repurposing

Although cancer treatment is still a major challenge, the discovery of new biotechnological weapons that take into consideration the patients’ genetic background, together with the advances in drug repurposing, a powerful strategy for the discovery of new cancer therapies, has allowed for the development of more precise cancer therapies in medicine.

Precision medicine has great potential and is a research area still under development that aims to rationally design treatment plans based on the patient’s characteristics, to allow for a better outcome for these patients [59,60]. To do so, it is important to focus on the drugs’ mechanism of action, as well as their molecular targets that may contribute to their anticancer effect.

One of the most relevant target cells in breast cancer treatment are the cancer stem cells (CSCs). These cells have a tumorigenic role, being responsible for tumour development, metastasis, recurrence, as well as the development of resistance mechanisms against several therapeutical approaches. Therefore, targeting CSCs could be an effective way to treat cancer. However, this is an extremely difficult task due to the heterogeneity of these cells, which supports the urgent need for further research about specific biomarkers of these cells [61]. This feature is only a small part of the complexity of the tumours, together with epigenetic, genetic, and proteomic aspects that reflect the heterogeneity of the outcomes associated with this disease.

To solve this problem, the key is targeting the cancer landscape using combinatory models of treatment, allowing for a multi-target effect. The combination of conventional anticancer agents with repurposed drugs could help increase the therapeutical efficiency, but sometimes this strategy is often associated with drug-drug interactions that can result in different outcomes and that must be carefully evaluated before designing combination therapies [62]. On the other hand, well-designed combination therapies can be a powerful pharmacological approach to overcome the genetic heterogeneity among cancer patients [63]. Indeed, several years ago, Law [64] and Frei [65] proposed the rational design of drug combinations assuming the premise that cancer cells within the same tumour (intratumoral heterogeneity) resistant to one drug might be killed by a second, different drug (and vice versa). Indeed, further studies have demonstrated that this is also true for interpatient heterogeneity and patients whose cancer did not respond to one drug had a chance of responding to a second, different drug [65,66,67]. Multiple studies have reported that combination therapies are more efficient than monotherapies in the treatment of different cancer types [68,69,70,71,72,73,74], but only a few of them relate the genetic background of the tumour cells with the action of the drugs, especially when studying repurposed drugs. Even with repurposed drugs in monotherapy, few studies explain the genetic mechanisms behind their mode of action. Most of the mechanisms explored when studying repurposed drugs are based on general carcinogenesis signalling pathways such as PI3K-AKT-mTOR and involve the study of DNA damage, apoptosis, or the study of the expression of important cancer-related proteins, such as p53 or Bcl-2 [75,76,77,78,79]. One of the exceptions is the recent study from Kumari et al., who performed a transcriptome analysis to understand the genes participating in artemisin anti-migratory and reduced invasive effects. Artemisin is a well-known antimalarial drug that exhibits potent anticancer effects in different types of cancer [80]. Studies like this allow us to understand if there is an up- or downregulation of important genes, such as tumour suppressor genes or oncogenes associated with growth-stimulating signalling pathways and relate these changes with the genetic predisposition of cancer patients.

**Figure 3:**
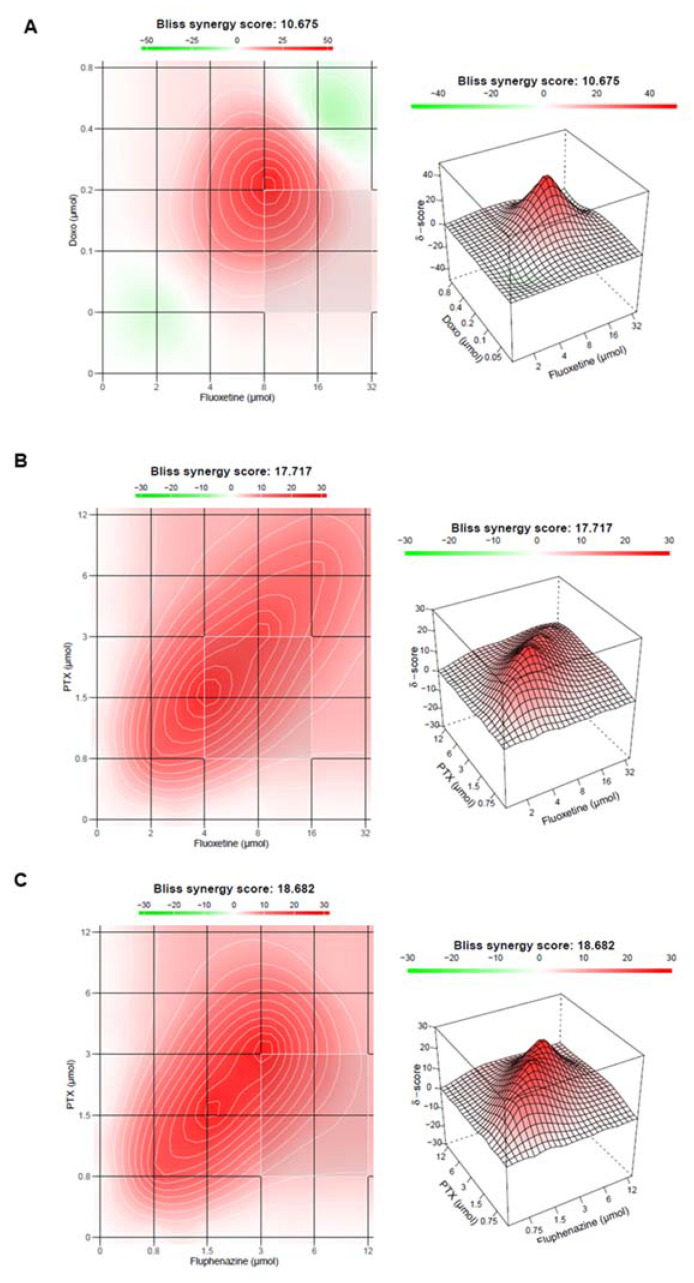
Bliss synergy plots representing the synergistic effects of (**A**) doxorubicin plus fluoxetine, (**B**) paclitaxel plus fluoxetine and (**C**) paclitaxel plus fluphenazine. Adapted from [82,83]. Synergy scores above 10 are considered synergic interactions.

Recent results from our research group have demonstrated that repurposed drugs belonging to antimalarial and central nervous system (CNS) classes show intrinsic antitumoral activity against MCF-7 breast cancer cells, an effect that is potentiated with the combination of antineoplastic agents [81,82,83]. More interestingly, we also found some of these drug pairs to change protein expression levels and to present synergistic interactions (Figure 3) but have not explored genetic alterations in the cells in the presence of these drugs. In this type of study, the genetic analysis could also be included to address the question of whether synergistic effects on the cell biological level also manifest at the genome level, in terms of synergistic genomic regulations. These findings will help to understand what genes and eventually novel targets are involved in combination treatments and relate them with the genetic background of patients, to personalize therapeutic regimens, achieve increased treatment efficacy and reduction of undesired side effects.

Another important aspect to increase the translational potential of repurposed drugs into the clinics is the considered design of clinical trials, in detail, the study design, which must scrutinize the different groups of cancer patients based on their genetic profile, allowing their stratification according to the predictive response to a certain drug to provide more adequate treatments and drug dosage administration to each patient group. It is also important to provide the correct follow-up of these cancer patients, to adjust dosages, if necessary, and better understand the treatment benefits or disadvantages, which could be the key to “speed up” the translational potential [25,59].

## 4. Conclusions

In conclusion, this review highlights the current progress made in the area of personalized cancer treatment based on patient tumour heterogeneity, considering the use of repurposed drugs and their associated benefits.

Globally, cancer is one of the leading causes of death, raising several public health and economic concerns. Cancer treatment faces several obstacles, which include the tumour cells’ development of mechanisms of resistance to the anticancer agents, decreasing the therapeutic efficacy of the available treatments. This supports the urgent need for the development of novel drugs or new strategies to combat cancer. Contrary to the *de novo* development, the drug repurposing is a time saving and low-cost approach for increasing the number of clinically available drugs to treat cancer, playing an important role in the design of more personalized treatments. Medicine of precision is a current hot topic concerning cancer treatment due to the heterogeneity associated with this disease.

In this article, we discussed how patient genetic variations could help to predict the different outcomes or even anticipate events of toxicity in response to a specific drug. Our findings reinforce that the search for new biomarkers that could be potential targets for the available drugs, linked to extensive investigation of the drugs’ pharmacokinetic and pharmacodynamic according to each patient’s tumour genetic profile, is urgent and essential to accelerate drug repurposing for personalized cancer treatments. To the best of our knowledge, this clinical application is limited and only if we invest more in well-designed clinical trials will we have significant progresses in this area. This involves the categorization of cancer patients into different groups based on their genetic particularities, based on the potential biomarkers associated with some of the repurposed drugs.

## Figures and Tables

**Table 1:** Some of the repurposed drugs used in breast cancer treatment and the associated biomarkers and the predictive response [29,31,33].

Drug	Biomarkers	Reported Outcomes
Doxorubicin	*CREB3L1*	Tumour sensitivity;
	*HLA* region	Cardiotoxicity predisposition.
Cyclophosphamide	*CYP2B6* and *CYP2C19*	Drug metabolic rate.
Everolimus	*CYP3A4*	Higher plasma concentration of everolimus.
	*ABCB1*	
	*PI3KR1*	Adverse Side Effects
	*RAPTOR*	
Tamoxifen	*CYP2D6*	Metabolic rate;
	*CYP19A1*	Effectiveness of the drug.
Anastrozole	*CSMD1*	Increased anastrozole sensitivity;
	*CYP19A1*	Drug response.
Paclitaxel	SNPs on *LPHN2*, *ROBO1*, *SNTG1* and *GRIK1*	Insensitivity to drug;bad prognosis
Aspirin	*PIK3CA* mutation	Drug Efficacy

## Data Availability

Not applicable.
